# Ineffective Learning Behaviors and Their Psychological Mechanisms among Adolescents in Online Learning: A Narrative Review

**DOI:** 10.3390/bs14060477

**Published:** 2024-06-06

**Authors:** Ji Li, Li Fang, Yu Liu, Jiayu Xie, Xiaoyu Wang

**Affiliations:** 1 West China School of Nursing, Sichuan University, Chengdu 610041, China; 1977liji_1226@scu.edu.cn; 2 Students’ Affairs Division, Sichuan Agricultural University, Chengdu 611830, China; 73222@sicau.edu.cn (L.F.); jiayuxie831@gmail.com (J.X.); 3 College of Psychology, Sichuan Normal University, Chengdu 610066, China; 20212093056@stu.sicnu.edu.cn; 4 West China Hospital, Sichuan University, Chengdu 610041, China

**Keywords:** online learning, inefficient learning behaviors, metacognition, adolescents

## Abstract

During the COVID-19 pandemic, many countries and regions experienced a surge in online learning, but the public complained about and questioned its effectiveness. One of the most important reasons for this was the inadequate metacognitive abilities of adolescents. Studies in learning sciences have identified various inefficient learning behaviors among students in online learning, including help abuse, help avoidance, and wheel spinning; all closely related to metacognition. Despite concerns about ecological validity, researchers in psychology have proposed the agenda-based regulation framework, the COPES model, and MAPS model, which may help explain the inefficient learning behaviors among adolescents in online learning. Future studies should aim to verify these theoretical frameworks within the context of online learning and elucidate the causes of inefficient learning behaviors; the design and optimization of online learning systems should be informed by theories in cognitive psychology.

## 1. Introduction

During the COVID-19 pandemic, many countries and regions experienced a surge in online learning. However, some parents, teachers, and students complained about and questioned the effectiveness of online learning [1]. The inadequate self-learning abilities of children and adolescents were identified as one of the crucial reasons for the suboptimal outcomes in online learning [2]. Represented by Flavell, the cognitive constructivist believes that self-learning is essentially metacognitive monitoring of learning, where students actively adjust learning strategies and effort based on their own learning abilities and the requirements of the learning task [3]. Unfortunately, children and adolescents often exhibit poor metacognitive abilities and are unable to effectively monitor and regulate their own learning behaviors, especially at a younger age, leading to ineffective learning behaviors. For example, children tend to be overly optimistic about their performance, displaying the overconfidence effect by overestimating their performance and placing themselves above others [4,5]. Such misjudgments lead learners to believe they have mastered the content, discouraging reflection after making mistakes and limiting seeking help from external sources. These ineffective learning behaviors have a severe negative impact on the effectiveness of online learning for youngsters, and metacognition is closely linked to this issue. Therefore, it is necessary to explore the manifestation and underlying mechanisms of ineffective learning behaviors in online learning from a metacognitive perspective, aiming to enhance students’ self-learning abilities, improve the quality of online learning, and transform students into expert learners.

In the field of learning science, psychologists excel in using true experiments to explore internal mechanisms, while researchers in computer science often use educational big data for machine learning modeling studies. Although different perspectives enrich the study of ineffective learning behaviors, the difficulty of dialogue between these fields has become increasingly apparent, resulting in a trend where the trees are seen but the forest is not. For instance, research on intelligent tutoring systems (ITS) often lags behind in its use of fundamental psychological theories compared to applied research. On one hand, ITS is highly practical, with data models focusing solely on predictive accuracy, while neglecting interpretability [6]. On the other hand, the urgent need for teaching drives researchers to propose numerous intervention strategies, mostly based on past teaching experience or system design experience, lacking theoretical guidance [7]. Therefore, this narrative review comprehensively discusses ineffective learning behaviors among adolescents and their psychological mechanisms in online learning environments from a metacognitive perspective, exploring the limitations of existing research and suggesting future research directions to address the current state of fundamental theory lagging behind applied research.

## 2. Ineffective Learning Behaviors among Adolescents in Online Learning

The behaviorist psychologist Skinner pioneered teaching machines in the 1950s [8]. Subsequently, digital learning environments gradually emerged, but early digital environments simply digitized instructional content, leaving learners passively receiving knowledge [8]. Intelligent learning environments are transformative products for digital education, with ITS being one of the most widely used intelligent learning environments, representing the application of artificial intelligence in education. ITS simulates one-on-one cognitive apprenticeship teaching by human teachers through a human–computer interaction interface [9]. Today, ITS is widely applied in various fields of teaching, such as medical education, mathematics, computer science, and reading and writing [10]. Its basic mode involves students inputting specified objects on the interactive interface, and the system’s teaching agent guides students step by step. For example, when solving an equation, the system provides an electronic canvas for learners to detail the calculation steps, known as substeps. The system guides students step by step in writing substeps to solve the equation through various human–computer interaction interfaces. In recent years, researchers have found various ineffective learning behaviors among children and adolescents in such systems, with the most typical being help avoidance, help abuse, wheel spinning, and gaming the system. While these behaviors may be related to metacognition [11,12], the lack of a systematic review weakens the theoretical foundation of educational data mining research.

### 2.1. Help Avoidance and Help Abuse

In ITS, each item/step is accompanied by hints or scaffolding of different granularity, which students can view at any time. The presentation order of hints goes from abstract to concrete, culminating in the final answer, aiming to facilitate students’ thinking and deepen learning. For example, in a problem like “What is the slope of the line graphed above?”, if the student answer incorrectly, the problem will be divided into a set of scaffolding items, which would ask students to complete each step required to solve the original problem. Substep1: “Slope is a number that measures the steepness of a straight line. We measure slope by picking 2 points and dividing the change along the y-axis by the change along the x-axis. What is the change along the y-axis for this line from point A to point B?”. Substep2: “What is the change in x for this line from point A to point B?”. If substep2 was answered correctly, “Good, Remember, the slope is the change in y divided the change in x. What is the slope for this line?” [13]. Despite providing carefully designed hints and scaffolding, students often fail to use them effectively [14]. They either ignore the hints or frequently click them to quickly obtain the final answer [15]. In fact, the students who need help the most are often the least willing to seek assistance. Therefore, examining students’ help-seeking behavior is crucial, as it may reflect their metacognitive level and help understand their academic failures, providing insights for optimizing and improving ITS.

In the practice and research into ITS, the two most common types of help-seeking deviations are help avoidance and help abuse. Help avoidance occurs when learners purposefully refrain from seeking assistance or support from the available resources, such as hints, explanations, or feedback provided by the educational system. Help abuse, on the other hand, involves an excessive reliance on or misuse of the available help features within the educational system [16,17]. Aleven et al. found that 36% of adolescent students showed help abuse and 19% exhibited help avoidance [16]. These help-seeking deviations were significantly negatively correlated with learning performance. For example, help avoidance was significantly negatively correlated with students’ transfer learning scores. Previous studies used Bayesian knowledge tracing algorithms to calculate learners’ probabilities of mastering a certain skill, where Kai et al. considered students as not having mastered a skill if the probability was below 0.6, marking students who did not seek help as having help avoidance [18]. Specifically, help avoidance manifested in two points: first, learners did not master the skill but did not click hints; second, students only had a slight understanding of the skill but did not consult the system’s glossary. Help abuse typically involved students who had not mastered a skill but continuously clicked hints or consulted the glossary, even when they had completely mastered the skill. Overall, more than 70% of ITS users in Aleven et al.’s study exhibited help-seeking deviations [16], indicating an urgent need to improve students’ metacognitive levels. Furthermore, these help-seeking deviations to some extent explained why online education is challenging. It places high demands on students’ metacognition and self-control, which many children and adolescents cannot meet.

### 2.2. Wheel Spinning

Persistence and self-control in learning are often significantly positively correlated with academic performance [18,19]. However, not all persistence is effective, and meaningless persistence may hinder learning and even lead to learned helplessness [18]. Beck and Gong first identified a type of meaningless persistence behavior among adolescent students in online learning, which they termed “wheel-spinning” [20]. It refers to learners spending a considerable amount of time on a learning topic without mastering it, neither reflecting nor seeking help, and persistently answering without reconsideration.

It is noteworthy that the definition of wheel-spinning varies among different learning systems, due to design differences. For instance, Matsuda et al. defined it as attempting a task more than five times without achieving mastery and without seeking help from the system. A machine learning model of wheel-spinning had a recall rate of 79%, but a precision of only 25% [21]. Flores and Rodrigo identified wheel-spinning as attempting a task more than five times without mastery and making four or more consecutive errors [16,17,18,19,20,21,22,23]. Similarly, ASSISTments considers answering three consecutive items correctly on the same skill as mastery [18], while Cognitive Tutor relies on Bayesian knowledge tracing to calculate the probability of learners mastering a specific skill, considering probabilities equal to or greater than 95% as mastery.

In summary, learners’ persistence in the learning process is meaningful, but there is a risk that this may transform into wheel-spinning and harming learning [24]. Therefore, a learning system capable of automatically distinguishing meaningful persistence from meaningless persistence behaviors (i.e., wheel-spinning) would be beneficial for providing timely feedback and conducting real-time interventions. Previous studies have attempted to differentiate between these types of persistence behaviors based on students’ actions and cognitive levels, such as students’ actions in the system or the number of practice attempts, as well as students’ mastery of knowledge points [18,25]. We argue that considering students’ metacognition is crucial, as it could potentially enhance the predictive effectiveness of machine learning models. Students’ decision to persist is often based on their metacognitive monitoring of their own knowledge mastery, i.e., metacognitive monitoring. Ineffective persistence by students may result from biased metacognitive monitoring, where students erroneously believe they can master the learning content without seeking external help.

### 2.3. Gaming the System

Gaming the system is defined as students leveraging the features and rules of online learning platforms to achieve success in a learning task without thoroughly contemplating the learning content [26]. For instance, students may engage in activities such as “flooding” the discussion forum, guessing correct answers to obtain higher scores, or intentionally quickly making errors [26,27,28]. These behaviors are prevalent among children and adolescence in online learning environments. Baker et al. suggested that gaming the system is related to learners’ self-regulated learning and metacognition [12]. Research indicates that students selectively engage in gaming behaviors; for example, some may avoid specific steps in a task, while others target areas where their mastery is weaker, and some may focus on well-mastered content [29]. Thus, gaming the system involves learners making behavioral decisions through cognitive monitoring.

Baker et al. categorized gaming the system into “Harmful gaming” and “Non-harmful gaming”. Non-harmful gaming behaviors primarily refer to students employing gaming behaviors in familiar and time-consuming problem-solving steps, allocating a significant amount of time to what they genuinely need to learn [12]. Alternatively, students may rapidly click hints to view answers and then engage in self-explanation or reflection [30]. However, both types of gaming behaviors are significantly correlated with poorer academic performance [12]. The present study tends to view gaming the system as an ineffective learning behavior resulting from learners’ cognitive monitoring bias. It is noteworthy that gaming the system is associated with emotional states and motivation; it may be perceived by students as a self-regulatory behavior to escape learning, especially when they experience boredom, confusion, or frustration [31].

## 3. Psychological Mechanisms of Ineffective Learning Behaviors in Online Learning

The various ineffective learning behaviors among children and adolescents mentioned earlier are closely related to metacognition, self-regulation, and self-control. Therefore, it is essential to review relevant theoretical models to explain the psychological mechanisms of ineffective learning behaviors. This paper focuses on Dunlosky et al.‘s agenda-based regulation (ABR) framework [32], Winne and Hadwin’s COPES theory [24,33], and Frazier et al.‘s MAPS model [34]. The reason for selecting these theories is that metacognitive monitoring plays a crucial role among these theories, with metacognitive monitoring being the core content of the ABR framework and COPES theory, while the MAPS theory emphasizes the control of metacognition by agency. In addition, they tend to focus more on a micro-level analysis compared to other concepts and theories, such the self-directed learning and community of inquiry framework. This is particularly evident given that the inefficient learning behaviors discussed in this paper are all examined through microanalytic measures of a temporal process (i.e., event measures), meaning that the analysis of these behaviors relies entirely on detailed log data from online learning systems. These theories can effectively guide future research in discovering factors contributing to the decline in students’ self-regulated learning performance and providing timely suggestions for improvement.

### 3.1. ABR Framework and Its Precursor Models

Before the ABR framework, Dunlosky and colleagues proposed several theoretical models for revealing the metacognitive mechanisms of learning time allocation. These precursor models laid the foundation for the ABR framework. They included the discrepancy-reduction model [35], the hierarchical model of self-regulated study [36], and the hypothesis of a region of proximal learning [37]. The discrepancy-reduction model focuses on the gap between the target state and the current state, leading learners to choose difficult items to narrow this gap [35,38]. The hierarchical model of self-regulated study constructs higher-level plans based on the target state and the current state, to optimize learning plans [36]. This model explains why learners may allocate less time to difficult items, where time-pressed learning groups tend to focus on simple items, while time-unconstrained groups lean towards difficult items [36]. However, these models still do not address the core issue of “dynamic learning processes”. Morehead et al. and Li et al. suggested that learners prioritize learning content they have not encountered or have not mastered [39,40]. This supports Metcalfe’s hypothesis of a region of proximal learning, where learners allocate time to unfamiliar and easily mastered items first, gradually increasing difficulty [37]. For example, experts allocate more time to difficult items than novices, and as proficiency deepens, the learner’s region of proximal learning gradually increases to a higher level. This indicates a close relationship between domain knowledge and the individual’s region of proximal learning, affecting learners’ time allocation, and reflects the dynamic changes in the learning process. Although research has closely related the region of proximal learning to domain knowledge, common measurement methods like self-reporting are inadequate for gauging changes in the region of proximal learning, making operationalization challenging. Additionally, while this hypothesis emphasizes the dynamic changes in learning, it does not specify the mechanisms of these changes or the impact of learner traits and external factors, etc., on the learning process.

**Figure 1:**
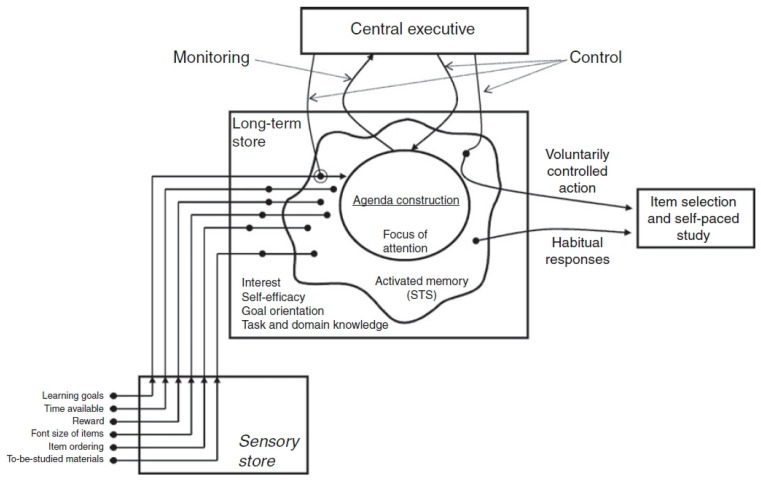
The agenda-based regulation (ABR) theoretical framework (adapted from [32]).

Therefore, researchers proposed the agenda-based regulation (ABR) theoretical framework (Figure 1). The ABR model suggests that learners construct learning agendas/plans based on various factors such as their characteristics and the nature of the task, to maximize learning goals [32]. Similarly to information processing, task-relevant internal and external stimuli activate long-term memory representations, then enter the working memory, triggering the learner’s attention to similar stimuli. The central executive system monitors and maintains attention focus, while inhibiting the influence of other irrelevant stimuli. If inhibition fails, learners will be dominated by habitual responses, making it difficult to complete the learning task.

According to the ABR framework, two main causes can lead to ineffective learning behaviors in online learning: (1) learners struggle to construct appropriate learning agendas or plans, and (2) the central executive system struggles to inhibit irrelevant stimuli. The ABR framework suggests that learners are influenced by various factors, such as their characteristics and the nature of the task, when constructing learning plans, but it does not specify how the construction of learning plans is influenced. Thus, our focus is primarily on the relationship between ineffective learning behaviors and the central executive system, such as monitoring bias, cognitive load, attention deficits, etc. For instance, help avoidance and wheel-spinning may be related to cognitive load; when learners’ cognitive resources are entirely focused on challenging tasks, they may lack additional cognitive resources to make help-seeking decisions [17]. Additionally, research has found that conspicuous system interface layouts (such as help-seeking signs) can make learners aware that they can seek help [17,41]. In this process, a conspicuous help-seeking sign serves as a perceptual cue, preventing learners from being influenced by habitual responses (such as help avoidance). However, the impact of perceptual cues on metacognitive monitoring is not always effective; one study found that learners tend to use perceptual cues that do not affect learning outcomes to monitor metacognitive processes, resulting in metacognitive errors [42]. We speculate that perceptual cues with low relevance to help seeking or learning outcomes may affect metacognitive monitoring processes, thereby promoting habitual responses (such as help avoidance).

Most of the precursor models of ABR framework focused on two aspects: item difficulty and time allocation, viewing the learning process as a static state, and considering the influence of other factors and dynamic changes in the learning process less. The ABR framework not only provides a possible explanation for ineffective learning behaviors, but also overcomes the limitations of task difficulty. It can more comprehensively consider other factors. Of course, the ABR framework also has its limitations, such as when the cognitive load or memory capacity is limited in the ABR framework, the mechanism of the central executive system will be affected accordingly, as well as how effectively it can inhibit irrelevant stimuli. Additionally, the factors that trigger habitual responses are not sufficiently explained, making interventions challenging.

### 3.2. COPES Model

**Figure 2:**
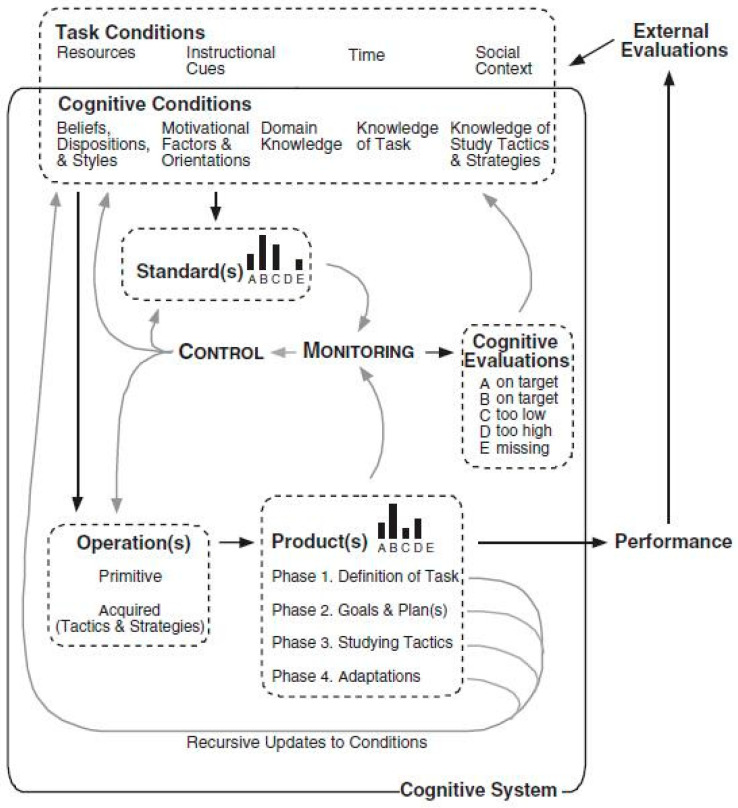
COPES model (adapted from [33]).

Winne and Hadwin proposed that learning occurs in four fundamental stages: task definition, goal setting and planning, studying tactics, and adaptation [33]. Each stage is influenced by both cognitive and metacognitive factors. They categorized factors interacting with these stages into five aspects: condition, operation, product, evaluation, and standard, collectively referred to as the COPES theoretical model (Figure 2). When learners receive task stimuli, they engage in cognitive operations (SMART processes) based on task-related conditions (internal conditions such as domain knowledge, learning strategies, motivation, and external conditions) and perceived environmental features. The cognitive operations involve forming definitions and standards for tasks, developing goals and plans based on declarative knowledge, and selecting appropriate learning strategies [43]. The content relevant to goals and the results of metacognitive evaluation generated throughout the cognitive process are collectively referred to as products.

Monitoring is one of the decisive conditions for self-regulated learning [44,45], and the COPES theory is no exception. Winne mentioned two dominant relationships in metacognitive evaluation: control and monitoring, and two hierarchical levels: meta-level and object-level [46]. For learners, the cognitive operations in the early stages of learning are exploratory, and with the accumulation of practical experience, the cognitive operation process becomes faster, gradually forming an automated cognitive pattern. If metacognitive monitoring detects differences between meta-level and object-level, metacognition will search for an effective cognitive form to modify the cognitive operations at the object level.

Taking wheel-spinning as an example, it can be considered an ineffective self-regulation behavior, and one of its underlying mechanisms may be a lack of metacognitive knowledge, making it difficult for learners to find effective cognitive patterns. They may produce results based on past experiences that cannot meet current learning requirements. For instance, compared to mixed training, learners in non-mixed training and control groups find it more difficult to transfer knowledge, and their metacognitive strategy levels are relatively lower [47,48]. This suggests that a lack of metacognitive knowledge may lead individuals to ineffectively apply a past cognitive pattern in a new environment. Secondly, Winne and Baker et al. found that, as the difficulty level of a task increases, learners’ metacognitive monitoring ability decreases [17,49]. This is likely because metacognitive monitoring struggles to identify differences between meta-level and object-level, thus maintaining the original cognitive operation. Motivation may play a crucial role in this; overly confident learners often have lower metacognitive monitoring levels [50].

Winne believes that motivation and emotions are essential regulatory factors that can influence the information of cognitive operations and metacognitive knowledge [43]. The behavior of gaming the system is likely an external manifestation of changes in learners’ motivation. Engelschalk et al. argued that when learners face tasks that are too difficult or too boring, they adjust their strategies for that specific task [51]. Additionally, Ocumpaugh et al. mentioned that when learners experience negative emotions such as boredom and frustration, they are likely to game the system [31]. This negative academic emotional experience may also lead students to avoid seeking help. For example, performance-oriented students may be reluctant to seek help when they do not understand the learning content or materials, fearing that seeking help will trigger negative reactions to their perceived abilities [52]. Help abuse can manifest in various ways (mastery vs. non-mastery), requiring a case-by-case analysis. We believe it is essential to first determine the motivation and goals of learners with help abuse, in order to further consider potential deviations in their overall self-regulation process.

In summary, the ABR framework seems more like a cognitive processing process that incorporates learner and task characteristics. Each step may cause cognitive overloads and an uneven distribution of cognitive resources, but it does not specify the impact of these cognitive-related factors on learning stages and behaviors. In comparison, the COPES model is relatively more comprehensive. It specifically outlines how stages and factors interact and creates possibilities for intervention. It may effectively explain the ineffective learning behaviors of the current study. On the one hand, it details the roles of self-regulated learning stages, cognition, and metacognition, helping us understand the learning process. On the other hand, it indicates the influence of motivation and academic emotions on cognition, metacognition, and learning behavior, which have been extensively discussed in the field of educational psychology.

### 3.3. MAPS Model

The MAPS model, proposed by Frazier et al., comprises three crucial aspects: possible self, metacognition, and agency [34]. Similar to Markus and Nurius’s concept of possible selves, the possible self in the MAPS model refers to the projection of the future—the goals one hopes to accomplish and the outcomes that one is concerned about [53]. These reflect the difference between the current self and the future self. This self-difference is a crucial motivation or resource for self-regulation and behavioral change. However, it does not inherently lead to behavioral change, but requires the implementation of intention to activate metacognition. Metacognition consists of metacognitive monitoring, metacognitive knowledge, metacognitive control, and metacognitive experience, where metacognitive control guides individual behavior, allowing individuals to adopt appropriate metacognitive strategies to achieve behavior control, all under the umbrella of metacognitive monitoring. Frazier et al. described these thought processes, motivations, and behaviors as a sense of agency, which receives internal perceptions of metacognitive monitoring for process attribution, outcome feedback, and attribution, thereby adjusting metacognition, behavior, and feeding back to the self-concept [34].

There may be two aspects of the MAPS model that lead to the failure of goal-directed behavior. One is related to metacognition, such as execution defects and the irrationality of metacognitive strategies [54,55]. For example, students who wheel-spin might maintain a behavior of unproductive persistence, due to the use of irrational metacognitive strategies. Despite the agency receiving and providing relevant information to the self-concept, the behavioral change may fail due to execution defects. Gaming the system is more related to motivation, and one possible reason for gaming is that students do not have the implementation intention to narrow their self-difference, resulting in non-activation of metacognition. The other aspect is related to agency, including agency perception and cognitive bias [56]. Gaming the system may also be a way for learners to sacrifice knowledge mastery for a sense of control over the learning task. For instance, the study by Bucknoff and Metcalfe indicated that participants were willing to trade less reward for more control [57]. Help avoidance and help abuse may be a result of students perceiving their agency as lower or higher than reality, leading to ineffective learning behaviors. However, this point deviates slightly from the MAPS theory; Frazier et al. believed that even if individuals overestimate their actual control level, it can still promote effective self-regulation [34].

In conclusion, the difference between the MAPS theory and the ABR framework and COPES theory lies in their emphasis on metacognition. The MAPS theory suggests that self-regulation does not revolve around metacognition but unfolds as regulatory activities based on self-concept and self-control, where individual behavioral performance stems from the possible self. The challenge lies in the macroscopic nature of the self-concept. It is unclear which experiences that learners gain during each self-regulation activity can update the possible self-state and how this state is manifested. Additionally, the MAPS model cannot focus on each self-regulation activity in every task process. It is challenging to determine when learners successfully update the self-state during self-regulation activities, and whether the updated self-difference prompts self-regulation to approach or move away from the current task. If learners do not update the self-state, does this mean that this self-regulation activity has failed? After failure, what coping strategies should learners adopt?

## 4. Limitations and Prospects

In summary, researchers have identified various ineffective learning behaviors among children and adolescents in online learning environments, such as help avoidance, help abuse, wheel-spinning, and gaming the system. These ineffective learning behaviors not only occur frequently but also show a moderate and significant negative correlation with students’ transfer learning, future learning readiness, and reduced test scores [17,58]. It is evident that students engaging in online learning face significant challenges in their metacognition, which we consider a crucial factor leading to the poor effectiveness of online learning.

Although previous studies have suggested a close connection between ineffective learning behaviors and metacognitive monitoring/regulation [12], there has been little research delving into the underlying psychological mechanisms. Moreover, online learning platforms have demonstrated its effectiveness to improve students’ academic performance. Exploring how to leverage these platforms to train learners’ metacognitive abilities is worth further investigation. This review detailed influential theories of self-regulated learning, explored the relationship between ineffective learning behaviors and metacognition, and attempted to integrate theory and practice, providing theoretical support for modeling and intervention studies. Several limitations also exist in this narrative review. Firstly, the ineffective learning behaviors reviewed herein primarily relied on empirical studies utilizing microanalytic measures of temporal processes (i.e., event measures). In other words, the analysis of these behaviors is entirely reliant on granular log data from online learning systems, closely associated with self-regulated learning. Consequently, our review perspective leant towards the analysis of micro-behaviors, and the theories included are more inclined towards process-oriented explanations. As a result, we may have overlooked a macro-theoretical perspective, such as neglecting the theoretical viewpoints of self-directed learning and the community of inquiry framework [59]. For example, the temporal and spatial separation between teaching and learning in online learning environments may lead to the absence of social presence and teaching presence, which could be one of the causes of ineffective learning behaviors in online learning. This highlights the importance for educators intentionally establishing social presence, teaching presence, and cognitive presence in online learning. Secondly, due to the reliance on studies using granular log data from online learning systems, there was limited attention given to Bloom’s higher-order taxonomy. Empirical research in the field finds it challenging to model students’ higher-order abilities using granular log data. Thirdly, given that this study provides a comprehensive discussion on ineffective learning behaviors with a relative broad scope, it is worth noting that this research is only a narrative review. Consequently, there might have been biases in the selection of literature, as the review lacked explicit inclusion and exclusion criteria and rigorous evaluation methods typically found in systematic reviews or meta-analysis.

Future studies should strengthen interdisciplinary research to consolidate and enrich cognitive psychology theories through educational data mining research, addressing the shortcomings of the low ecological validity in cognitive psychology [60]. Conversely, cognitive psychology experiments could compensate for the drawbacks of practicality over theory in educational data mining research, particularly in cases where the design of online learning platforms lacks support from cognitive psychology theories. The COPES model holds the potential to explain the ineffective learning behaviors among children and adolescents in online learning, but future research should investigate its applicability in intelligent tutoring environments, utilizing real instructional materials, log data from online platforms, and experimental techniques from cognitive psychology. Finally, regarding machine learning studies on ineffective learning behaviors, previous researchers have mostly considered learners’ cognitive and behavioral features [58,61,62,63], relatively neglecting metacognition, which may affect the predictive accuracy of machine learning models. Future research could shift the focus of feature engineering onto metacognition, combined with physiological indicators from psychology, such as EEG and eye-tracking, aiming to enhance the model performance of ineffective learning behaviors and lay a solid foundation for personalized interventions.

## Data Availability

Not applicable.

## References

[1] Mihova P., Stankova M., Andonov F., Tsoukka K., Proedrou A., Tsetsila E., Alshawesh H., Mavrothanasi M., Stoyanov S., Uskov V.L., Howlett R.J., Jain L.C. (2022). Parental attitudes towards online learning—Data from four countries. Smart Education and E-Learning—Smart Pedagogy.

[2] Dong C., Cao S., Li H. (2020). Young children’s online learning during COVID-19 pandemic: Chinese parents’ beliefs and attitudes. Child. Youth Serv. Rev..

[3] Wang F., Yang X., Huang H. (2022). Research summary on meta-cognitive theory and practice of China. Res. Pract. High. Educ..

[4] Butler G., McManus F. (2014). Child Psychology: A Very Short Introduction.

[5] Dunning D., Griffin D.W., Milojkovic J.D., Ross L. (1990). The overconfidence effect in social prediction. J. Personal. Soc. Psychol..

[6] Carvalho D.V., Pereira E.M., Cardoso J.S. (2019). Machine learning interpretability: A survey on methods and metrics. Electronics.

[7] Molennar I., Horvers A., Dikstra R., Baker R. (2019). Designing dashboards to support learners’ self-regulated learning. Proceedings of the 9th International Conference on Learning Analytics and Knowledge.

[8] Huang R., Yang J., Hu Y. (2012). From digital to smart: The evolution and trends of learning environment. Open Educ. Res..

[9] Liang Y., Liu C. (2018). The application status, typical characteristic and development trends of artificial intelligence in education. China Educ. Technol..

[10] Gao H., Long Z., Liu K., Xu S., Cai Z., Hu X. (2016). AutoTutor: Theories, technologies, applications and potential impacts. Open Educ. Res..

[11] Beck J., Rodrigo M., Mercedes T. (2014). Understanding wheel spinning in the context of affective factors. Intelligent Tutoring Systems, Proceedings of the 12th International Conference on Intelligent Tutoring Systems, Honolulu, HI, USA, 5–9 June 2014.

[12] Baker R.S., Corbett A.T., Roll I., Koedinger K.R., Aleven V., Cocea M., Hershkovitz A., de Caravalho A.M., Mitrovic A., Mathews M., Azevedo R., Aleven V. (2013). Modeling and studying gaming the system with educational data mining. International Handbook of Metacognition and Learning Technologies.

[13] Razzaq L., Heffernan N.T., Ikeda M., Ashley K.D., Chan T.W. (2006). Scaffolding vs. hints in the Assistment system. Intelligent Tutoring Systems.

[14] Duong H., Zhu L., Wang Y., Heffernan N.T. A prediction model that uses the sequence of attempts and hints to better predict knowledge: “Better to attempt the problem first, rather than ask for a hint”. Proceedings of the 7th International Conference on Educational Data Mining.

[15] Price T.W., Liu Z., Cateté V., Barnes T. Factors influencing students’ help–seeking behavior while programming with human and computer tutors. Proceedings of the 2017 ACM Conference on International Computing Education Research.

[16] Aleven V., McLaren B., Roll I., Koedinger K. (2006). Toward meta–cognitive tutoring: A model of help seeking with a Cognitive Tutor. Int. J. Artif. Intell. Educ..

[17] Almeda V., Baker R., Corbett A. (2017). Help avoidance: When students should seek help, and the consequences of failing to do so. Teach. Coll. Rec..

[18] Kai S., Almeda M.V., Baker R.S., Heffernan C., Heffernan N. (2018). Decision tree modeling of wheel–spinning and productive persistence in skill builders. J. Educ. Data Min..

[19] Credé M., Tynan M.C., Harms P.D. (2017). Much ado about grit: A meta-analytic synthesis of the grit literature. J. Personal. Soc. Psychol..

[20] Beck J.E., Gong Y. (2013). Wheel–spinning: Students who fail to master a skill. International Conference on Artificial Intelligence in Education, Proceedings of the 16th International Conference of Artificial Intelligence in Education, Memphis, TN, USA, 9–13 July 2013.

[21] Matsuda N., Chandrasekaran S., Stamper J. How quickly can wheel spinning be detected. Proceedings of the 9th International Conference on Educational Data Mining.

[22] Schneider M., Preckel F. (2017). Variables associated with achievement in higher education: A systematic review of meta-analyses. Psychol. Bull..

[23] Flores R.M., Rodrigo M.M.T. (2020). Wheel–Spinning Models in a Novice Programming Context. J. Educ. Comput. Res..

[24] Palaoag T.D., Rodrigo M.M.T., Andres J.M.L., Andres J.M.A.L., Beck J.E. Wheel-spinning in a game-based learning environment for physics. Proceedings of the 13th International Conference on Intelligent Tutoring Systems.

[25] Owen V.E., Roy M.H., Thai K.P., Burnett V., Jacobs D., Keylor E., Baker R.S. Detecting Wheel-Spinning and Productive Persistence in Educational Games. Proceedings of the 12th International Conference on Educational Data Mining.

[26] Nunes T.M., Bittencourt I.I., Isotani S., Jaques P.A. Discouraging gaming the system through interventions of an animated pedagogical agent. Proceedings of the 11th European Conference on Technology Enhanced Learning.

[27] Cheng R., Vassileva J. Adaptive reward mechanism for sustainable online learning community. Proceedings of the 12th International Conference on Artificial Intelligence in Education.

[28] Paquette L., Baker R.S., Moskal M. A system–general model for the detection of gaming the system behavior in CTAT and LearnSphere. Proceedings of the International Conference on Artificial Intelligence in Education.

[29] Baker R.S., Corbett A.T., Koedinger K.R., Wagner A.Z. Off-task behavior in the cognitive tutor classroom: When students “game the system”. Proceedings of the ACM CHI 2004 Conference on Human Factors in Computing Systems.

[30] Rus V., Banjade R., Niraula N., Gire E., Franceschetti D., Popescu E., Kinshuk, Khribi M.K., Huang R., Jemni M., Chen N.-S., Sampson D.G. (2017). A study on two hint-level policies in conversational intelligent tutoring systems. Innovations in Smart Learning.

[31] Ocumpaugh J., Andres J.M., Baker R., DeFalco J., Paquette L., Rowe J., Mott B., Lester J., Georgoulas V., Brawner K. Affect dynamics in military trainees using vMedic: From engaged concentration to boredom to confusion. Proceedings of the 18th International Conference on Artificial Intelligence in Education.

[32] Dunlosky J., Ariel R. (2011). Self-regulated learning and the allocation of study time. Psychol. Learn. Motiv..

[33] Winne P.H., Hadwin A.F., Dunlosky H., Dunlosky J., Graesser A. (1998). Studying as self-regulated learning. Metacognition in Educational Theory and Practice.

[34] Frazier L.D., Schwartz B.L., Metcalfe J. (2021). The MAPS model of self-regulation: Integrating metacognition, agency, and possible selves. Metacognit. Learn..

[35] Dunlosky J., Hertzog C., Dunlosky H., Dunlosky J., Graesser A. (1998). Training programs to improve learning in later adulthood: Helping older adults educate themselves. Metacognition in Educational Theory and Practice.

[36] Thiede K.W., Dunlosky J. (1999). Toward a general model of self–regulated study: An analysis of selection of items for study and self-paced study time. J. Exp. Psychol. Learn. Mem. Cogn..

[37] Metcalfe J. (2009). Metacognitive judgments and control of study. Curr. Dir. Psychol. Sci..

[38] Liu X., Fang G., Yang X. (2004). The review of the studies about allocation of study time overseas. Adv. Psychol. Sci..

[39] Morehead K., Dunlosky J., Foster N.L. (2017). Do people use category-learning judgments to regulate their learning of natural categories. Mem. Cogn..

[40] Li P., Zhang Y., Li W., Li X. (2018). Age-related differences in effectiveness of item restudy choices: The role of value. Aging Neuropsychol. Cogn..

[41] Roll I., Baker R.S., Aleven V., Koedinger K.R. (2014). On the benefits of seeking (and avoiding) help in online problem-solving environments. J. Learn. Sci..

[42] Chen F., Li F., Li W. (2016). Effects of perceptual cues on metamemory monitoring and control. Adv. Psychol. Sci..

[43] Winne P.H. (2022). Modeling self-regulated learning as learners doing learning science: How trace data and learning analytics help develop skills for self-regulated learning. Metacognit. Learn..

[44] Zimmerman B.J., Boekaerts M., Pintrich P., Zeidnerm M. (2000). Attaining self-regulation: A social cognitive perspective. Handbook of Self-Regulation.

[45] Zimmerman B.J., Zimmerman B.J., Schunk D. (2001). Theories of self-regulated learning and academic achievement: An overview and analysis. Self-Regulated Learning and Academic Achievement: Theoretical Perspectives.

[46] Winne P.H., Zimmerman B.J., Schunk D.H. (2017). Cognition and metacognition within self-regulated learning. Handbook of Self-Regulation of Learning and Performance.

[47] Schuster C., Stebner F., Leutner D., Wirth J. (2020). Transfer of metacognitive skills in self-regulated learning: An experimental training study. Metacognit. Learn..

[48] Leopold C., Leutner D. (2015). Improving students’ science text comprehension through metacognitive self-regulation when applying learning strategies. Metacognit. Learn..

[49] Winne P.H., Zimmerman B.J., Schunk D.H. (2001). Self–regulated learning viewed from models of information processing. Self-Regulated Learning and Academic Achievement: Theoretical Perspectives.

[50] Miller T.M., Geraci L. (2014). Improving metacognitive accuracy: How failing to retrieve practice items reduces overconfidence. Conscious. Cogn..

[51] Engelschalk T., Steuer G., Dresel M. (2016). Effectiveness of motivational regulation: Dependence on specific motivational problems. Learn. Individ. Differ..

[52] Karabenick S.A. (2004). Perceived achievement goal structure and college student help seeking. J. Educ. Psychol..

[53] Markus H., Nurius P. (1986). Possible selves. Am. Psychol..

[54] Metcalfe J., Metcalfe J., Herberts T. (2013). “Knowing” that the self is the agent. Agency and Joint Attention.

[55] Metcalfe J., Mischel W. (1999). A hot/cool-system analysis of delay of gratification: Dynamics of willpower. Psychol. Rev..

[56] Zalla T., Miele D., Leboyer M., Metcalfe J. (2015). Metacognition of agency and theory of mind in adults with high functioning autism. Conscious. Cogn..

[57] Bucknoff Z.J., Metcalfe J., Cleary A., Schwartz B. (2020). Memory under the SEA (Subjective Experience of Agency). Memory Quirks.

[58] Baker R.S., Andrew W.B., Sujith M.G., Zhang S., Hawn A. (2020). Predicting k-12 dropout. J. Educ. Stud. Placed Risk (JESPAR).

[59] Garrison D.R., Arbaugh J.B. (2007). Researching the community of inquiry framework: Review, issues, and future directions. Internet High. Educ..

[60] Hatfield G. (2002). Psychology, philosophy, and cognitive science: Reflections on the history and philosophy of experimental psychology. Mind Lang..

[61] Crossley S.A., Karumbaiah S., Ocumpaugh J., Labrum M.J., Baker R.S. (2020). Predicting math identity through language and click-stream patterns in a blended learning mathematics program for elementary students. J. Learn. Anal..

[62] Zhang J., Andres J., Hutt S., Baker R.S., Ocumpaugh J., Nasiar N., Mills C., Brooks J., Sethuaman S., Young T. (2022). Using machine learning to detect smart model cognitive operations in mathematical problem-solving process. J. Educ. Data Mining.

[63] Hutt S., Wong A., Papoutsaki A., Baker R.S., Gold J.I., Mills C. (2024). Webcam-based eye tracking to detect mind wandering and comprehension errors. Behav. Res. Methods.

